# The Influence of Stinging Nettle (*Urtica dioica* L.) Infusions on the Techno-Functionality of k-Carrageenan Hydrogels

**DOI:** 10.3390/gels12040313

**Published:** 2026-04-07

**Authors:** Andreea Pușcaș, Cristian Szekely, Flavius George Viorel, Alexandra Raluca Lazăr, Anda Elena Tanislav, Andruța Elena Mureșan, Vlad Mureșan

**Affiliations:** Food Engineering Department, Faculty of Food Science and Technology, University of Agricultural Sciences and Veterinary Medicine Cluj-Napoca, 400372 Cluj-Napoca, Romania; andreea.puscas@usamvcluj.ro (A.P.);

**Keywords:** hydrogels, stinging nettle (*Urtica dioica* L.) infusions, antioxidant potential

## Abstract

In the current study, bioactive-loaded hydrogels were developed with k-carrageenan (1%), and water was replaced with infusions of *Urtica dioica* L., which modulated the polymer chains to create more robust networks. *Urtica dioica* L. infusions were obtained with different infusion durations (5 or 10 min) or plant-to-water ratios (0.4, 1, or 2 g/100 mL). The hydrogels were characterized for stability by assessing the syneresis rate and textural and rheological attributes. To elucidate the influence of the infusion on the mechanisms of k-carragenan, temperature ramp tests were applied and FTIR spectra were acquired. Replacing water with *Urtica dioica* L. infusions for obtaining k-carrageenan hydrogels led to lower syneresis rates (3.34 ± 0.03% and 6.67 ± 0.33%), while the hydrogels showed increased hardness, but lower resilience and cohesiveness. The rheological parameters confirmed the reinforcement; higher G′ and gelling temperatures were registered compared to the reference. While FTIR spectra showed that the primary chemical backbone remained intact, the physicochemical changes indicate a strong physical synergy between nettle polyphenols and the κ-carrageenan chains. Of all samples, the highest antioxidant potential value of 94.66% was exhibited by the infusion obtained in 15 min with a ratio of plant material of 2/100 g. These findings demonstrate that plant-to-water ratios and infusion times are critical parameters for tuning the physical properties and biological efficacy of hydrogels for medical or food applications.

## 1. Introduction

Nettle (*Urtica dioica* L.) is considered one of the most prevalent spontaneous perennial herbs in the world, but because of its numerous health-related effects and requirements, it has also been cultivated in plantations. After harvesting, the plant exhibits stability issues; thus, processing and conditioning are required to preserve its beneficial potential. Processing involves sun drying, shadow drying, oven drying or even freeze drying. Dried stinging nettle leaf powders are commonly used for infusion or decoction preparation, due to their medicinal and nutritional effects, as they are efficient in combating diseases such as anemia, urinary tract infections, arthritis, and obesity [1,2]. Nettle leaves have a complex chemical composition including proteins, amino acids, fatty acids, organic acids, minerals, polysaccharides and bioactive compounds [3]. Nettle is known to possess antioxidant properties due to its constituents, such as the carotenoid compounds, vitamins (A and C), flavonoids and phenolic compounds [3]. However, the drying method and higher temperatures or longer durations could denature these bioactive compounds. Moreover, the maturity stage of the plant and the leaves was shown to impact the bioactive compounds, with immature nettle leaves being more abundant in polyphenols [4]. Less β-carotene was detected in decoctions from dried nettles than in infusions, given the compound’s polarity and the temperature exposure. It was demonstrated that subcritical and microwave extraction approaches preserve the bioactive potential of the plants. However, these procedures require specialized equipment, which can be relatively expensive, especially in comparison with conventional extraction techniques [5,6]. Research evaluating the *U. dioica* uses gained popularity, with the number of available studies growing tenfold in the last 10 years [2]. Nettle extracts or leaves were used for developing functional foods, due to their bioactive compounds, as follows: bread [7] and cookies [8] enriched in phenolic compounds and minerals, ice cream enriched with phenols [9]; pasta with pigments, increased fiber and mineral contents [10]; antimicrobial potential for bioactive edible films production [11] and vegetable coagulant for cheese production [12]. Nettle juice gel was studied as a treatment for hair loss-related diseases, such as *Telogen effluvium* [13]. Hydrogels were recently developed from nettle extracts to be used in wound treatments [14,15].

Infusion can be obtained if the plant material is suspended/soaked/steeped in hot or boiled water for a specified time, and the polar components could be extracted in the aqueous media, including organic acids, phenolic acids, flavonoids, amino acids, vitamins, and minerals. In households, the infusing duration is around 5 min [16], while for Stinging Nettle, an optimal infusion duration of 10 min was established when in a relationship with a higher content of bioactive compounds or antioxidant potential in a previous study [17]. Previous studies were focusing on the conditioning of the plant or the infusion temperature, while in the present study, besides the duration and ratio of the plant material, the water was also varied to explore the efficiency of the process. Hydrogels are generally prepared by converting hydrophilic polymer solutions into a 3D network structure via physical or chemical crosslinking. Carrageenan (classified as λ, κ, ι, ν, θ, and μ, based on the sulfate content) is obtained from red marine algae of the Rhodophyceae class and has been used in the food industry as an ingredient for manufacturing puddings, cottage cheese, desserts, or sausages [18]. K-carragenan usually works based on chemical crosslinking of the sulfate groups with cations such as Ca^2+^, Na^+^ or K^+^, but has been reported to also generate hydrogels due to physical crosslinking (with electrolyte solutions) [19]. Recently, tender coconut water was studied as an alternative to potassium chloride (KCl) to crosslink κ-carrageenan hydrogels loaded with diclofenac sodium stabilized using polysorbate-80, to increase its hydrophilicity, because coconut water contains 250–300 mg of K/100 g, demonstrating that natural alternatives could be used to ensure crosslinking [19]. K-carrageenan films loaded with different polyphenol extracts were already designed to be used in the food industry and demonstrated excellent release of the polyphenolic compounds from film to the packaged foods, as well as the ability to act as a pH-sensitive label [20,21]. Hydrogels could be further used in designing low caloric foods, in sports nutrition or in molecular gastronomy, or for obtaining products with modified fat systems. One of the most studied applications of these structures is their usage as packaging materials containing biologically active substances that are capable of preventing and indicating food spoilage [22].

The current study proposes the development of k-carrageenan-based hydrogels from the *U. dioica* L. infusions that were obtained with different brewing durations or plant-to-water ratios, as systems loaded with bioactive compounds. The obtained hydrogels were characterized for stability by assessing the syneresis rate and the textural and rheological attributes. Moreover, to elucidate the influence of the infusion composition on the mechanisms of k-carragenan, temperature ramp tests were applied. FTIR spectra of the samples were acquired and the antioxidant potential of the samples was spectrophotometrically assessed.

κ-carrageenan hydrogels were previously designed as bioactive compound carriers and loaded with both hydrophilic (anthocyanins, betacyanins) [23] and hydrophobic substances (curcumin) [24,25], but the current method proposes structuring the infusions that are abundant in health-promoting agents, such as polyphenols, pigments, and organic acids. The hydrogels characterized in the present study would be of high value for medical or food applications.

## 2. Results and Discussion

### 2.1. Results Regarding the Infusions

L, a* and b* color values were significantly affected by the infusion duration and ratio (Table 1). The infusions presented a dark appearance, with low values of L varying from 7.90 ± 1.10 to 27.21 ± 0.78; higher L values were obtained with a shorter duration of infusion, suggesting that lower amounts of water-soluble compounds were extracted. Two-way ANOVA showed significant main effects of the material-to-water ratio (F = 133.12, *p* < 0.0001) and infusion/brewing duration (F = 449.61, *p* < 0.0001), and a significant A × B interaction (F = 154.47, *p* < 0.0001). Because the interaction was significant, simple effects analyses were conducted, and no statistical significant differences were obtained between samples obtained from 2/100 g, regardless of the brewing duration, in terms of lightness (L value). The infusing duration affected L for samples with a lower material-to-water ratio.

In terms of a*, the parameter varied between 5.09 ± 0.42 and 13.41 ± 1.54, while the samples had a dark green aspect. Samples brewed for 5 min differed statistically, due to the material-to-water ratio used to obtain emulsions. The infusing duration did not affect the a* and b* color parameter of the infusions obtained from 2 g material/100 g water.

The b* parameter varied between 5.84 ± 0.48 and 10.51 ± 0.38, with higher values being obtained for the b* parameters of samples with an infusing duration of 15 min.

The effect of using fresh or oven-dried stinging nettle leaves (*Urtica dioica* L.) on the infusions was previously studied by T.T. Shonte, 2017 and H.L. de Kock, 2020, who revealed that dried samples led to improved flavor and aroma and differences in the color of the infusions [3,26]. Oven-dried nettle leaves (70 °C/15 h) were reported to possess a higher total phenolic content than freeze-dried or fresh samples [26].

In another study, after the first brew of nettle dried at 70 °C/15 h, the infusion presented the following parameters: L = 49.70, a* = −6.5 and b* = 25.7. However, those from fresh nettle were as follows: L = 33.30, a* = −7.4 and b* = 12.90 [3]. Lower L and b* values were determined in the current study for each type of infusion, regardless of the infusing duration and plant-to-water ratio; thus, the drying protocol could be a decisive factor for the amount of bioactive compounds present in the plant material, since the drying method has a major impact on nutrient degradation or retention. Lower pH values were determined for infusions brewed for 5 min compared to those obtained in 15 min, but the ratio of solid material/100 g water also influenced the pH; the differences were not that high, as seen in Table 1.

### 2.2. Syneresis Rates and Textural Properties of Hydrogels

**Figure 1:**
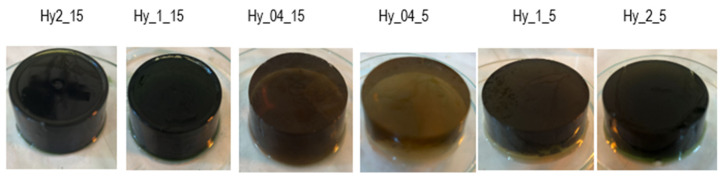
Hydrogels obtained from infusions of dried nettle leaves—codification and appearance.

The hydrogel samples are codified depending on the infusion used for their manufacture, and their appearance is shown in Figure 1.

An insufficiency of k-carrageenan hydrogels is that water leaks out from the hydrogel during storage, resulting in increased syneresis rates. In another study, a syneresis rate of 10% was reported for a 1% carrageenan hydrogel after 24 h and 20% after 48 h, with the rate varying with the amount of carrageenan employed for structuring [27]. A similar result was obtained for the reference in the current study, with a syneresis rate of 9.73 ± 1.56% after 24 h, while replacing water with (*Urtica dioica* L.) infusions led to lower syneresis rates, ranging between 3.34 ± 0.03 and 6.67 ± 0.33% (Table 2) in the first 24 h of the follow up. However, after 72 h, the water leakage of the systems was more prominent, leading to increased syneresis rates of up to 14.35 ± 2.46%, which was still lower than what was previously reported. This was achieved by Hy_04_15, while the reference registered a syneresis rate of 12.53 ± 2.74. However, the follow up of the syneresis rates has a limitation for practical food or functional material applications, with longer-term stability assessment (≥7 days) being necessary. However, hydrogels could be included in other structures like emulgels or bigels, which could gather higher stability.

The addition of 5% erythritol was reported to lower syneresis rates of peach κ-carrageenan edible gels [28]. Besides the polysaccharide concentration, the pH and salt presence also influence this, but it was stated that for stronger gels, the contractions which expel the water were diminished [29]. The elasticity of the k-carrageenan hydrogels is strongly influenced by the temperature [29]. The results of our study reconfirm these findings, since infusion-based hydrogels exhibit increased hardness compared to the reference and lower syneresis rates. Samples syneresis after 72 h were statistically similar, except for Hy_04.15 and the reference; thus, despite improvements after the first 24 h, the stability is then affected by the extracted substances.

The increased hardness of the structures might be due to the organic acids present in the infusions, whose presence were confirmed by the values registered for the pH, but also by some peaks in the FTIR spectra, specific to organic acids. Hy_1_5 presented the highest value of hardness, which was statistically different from the rest of the samples, while Hy1_15 and the reference were the lowest, highlighting the importance of exploring different extraction durations for tuned textural parameters. Hardness, along with the syneresis rates and the rest of the textural parameters (resilience, cohesiveness and gumminess), is summarized in Table 2.

Peach juice solution (containing 0.2% KCl) with 0.9% k-carrageenan led to the formation of a jelly with a hardness of 31.01 ± 3.32 N, a cohesiveness of 0.33 ± 0.15, and a higher gumminess of 11.44 ± 5.94 N.

Thus, the compounds present in the peach juice (sugar, organic acids, dietary fiber, phenolic compounds, vitamin, minerals, carotenoids, phenolic compounds) and also the erythritol or KCl improve the structure of k-carrageenan gels [30]. Resilience, or the elastic recovery of the sample, is a measure of a hydrogel’s ability to recover after deformation, and it was higher for the reference than for the infusion-based hydrogels, which presented much lower values. The resilience of Hy_1_15 and Hy_2_15 was the lowest. A higher ratio of plant material and longer infusion durations lead to decreased resilience; however, Hy_04_15 and hydrogels with 5 min infusions maintained higher resilience values, but they were lower than that of the reference (0.41 ± 0.04). The cohesiveness registered for the samples obtained from infusions were similar, but lower than that of the reference. Hy_1_15 and Hy_2_15 also had the lowest cohesiveness among the samples.

In another study, a 2% k-carrageenan hydrogel was reported to have a cohesiveness of 0.65 and gumminess close to 10 N, which is consistent with our results [31]. Gumminess is a measure of force that is required to disintegrate a semi-solid hydrogel during compression, which is obtained by multiplying the hardness and cohesiveness. Increased gumminess was determined for samples which exhibited higher hardness, but also for Hy_04_15, which presented low hardness values (16.74 ± 1.33 N), but the highest cohesiveness among the hydrogels was obtained from infusions. The Hy_04_15 sample’s gumminess was similar to that of the reference, whereas the rest of the samples presented lower values.

### 2.3. Rheology

**Figure 2:**
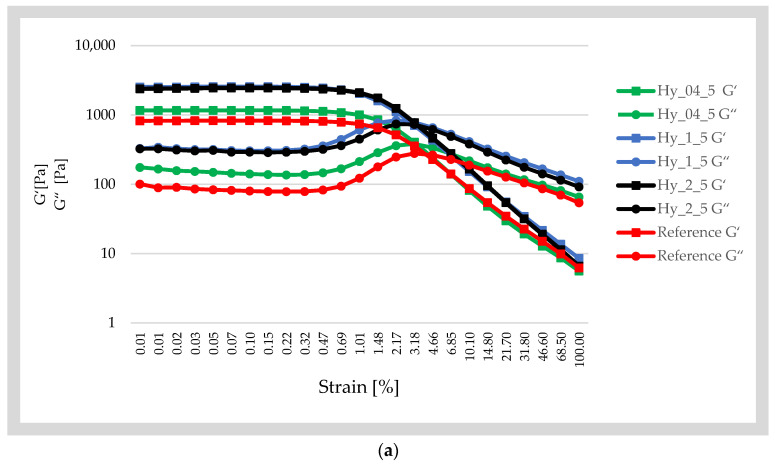

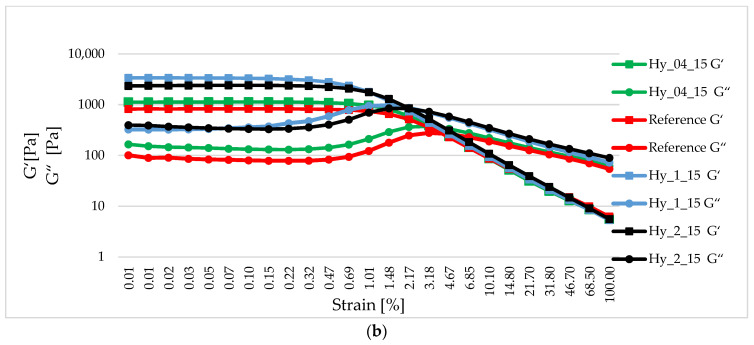
Influence of infusion duration (**a**)—5 min and (**b**)—10 min and the plant:water ratio of infusions on the plastic (G′) and elastic (G″) modulus of the 1% k carrageenan hydrogels.

During the experiment, all the samples showed a clear LVR stage, during which G′ and G″ remained almost constant (Figure 2), followed by changes in both moduli that produced a crossover between G′ and G″, and a final step in which both moduli decreased slowly as the structure was destroyed. Infusion-based samples led to increases in G′, compared to the reference. G′_LVR_ was computed by the software and occurred between 0.1 and 0.5% strain. For the reference, the plateau occurred at 0.5 ± 0.004, with a G′_LVR_ of 798.18 ± 127.73 Pa, while the highest G′_LVR_ was recorded for Hy_1_15 (G′_LVR_ = 3278.45 ± 394.28 Pa) at 0.1%. The ratio of the nettle and infusing durations influenced the storage modulus values; Hy_1_15 and Hy_1_5 showed the highest G′ at the beginning of the experiment, 3383.9 ± 400.93 Pa and 2534 ± 159.03 Pa, respectively. Hy_1_15 presented the lowest loss factor (0.0955 ± 0.009), hence the highest elastic behavior, while this sample also registered low values of cohesiveness. However, the reference reported G′ values that were three times lower (820.23 ± 157.24 Pa), despite the high values of the cohesiveness.

K-carrageenan was reported to increase the viscoelastic properties, rigidity or plasticity of gels due to the hydrogen bonds, while the presence of hydrophilic compounds extracted in the infusions prepared with a ratio of 1:100 mL or 2:100 mL with infusing durations of 5 or 15 min led to stronger networks, and thus possibly more hydrogen bonds. This is confirmed by the FTIR spectra of the samples, which registered lower absorbance of the peaks formed in the –OH corresponding regions.

The crossover points (G′ = G″) of Hy_1_15 and Hy_2_15 occurred first at 2.17%, while Hy_1_5 and Hy_2_5 presented structure deformation at 3.18%. The reference and Hy_0.4_5 presented higher resistance to deformation and crossover points around 4.66%; thus, this is a cross-linked matrix.

Since the shear thinning effect occurred after the G crossover point, in the 0.466–100% region, the power-law model parameters were gathered to observe the G′ of the samples. The highest consistency index, K, ranged between 15.44 and 19.95 and was the highest for Hy_2_5 and Hy_1_5, but Hy_2_15 and Hy_1_15 registered statistically similar values. The reference had the lowest K value, but also the lowest plastic modulus. Infusions with higher plant-to-water ratios led to higher consistency index values for the corresponding hydrogels (Table 3). The samples exhibited non-Newtonian behavior (*n* < 1), which was the lowest for Hy_2_5 and Hy_1_15 and more dominant for the reference hydrogel.

**Figure 3:**
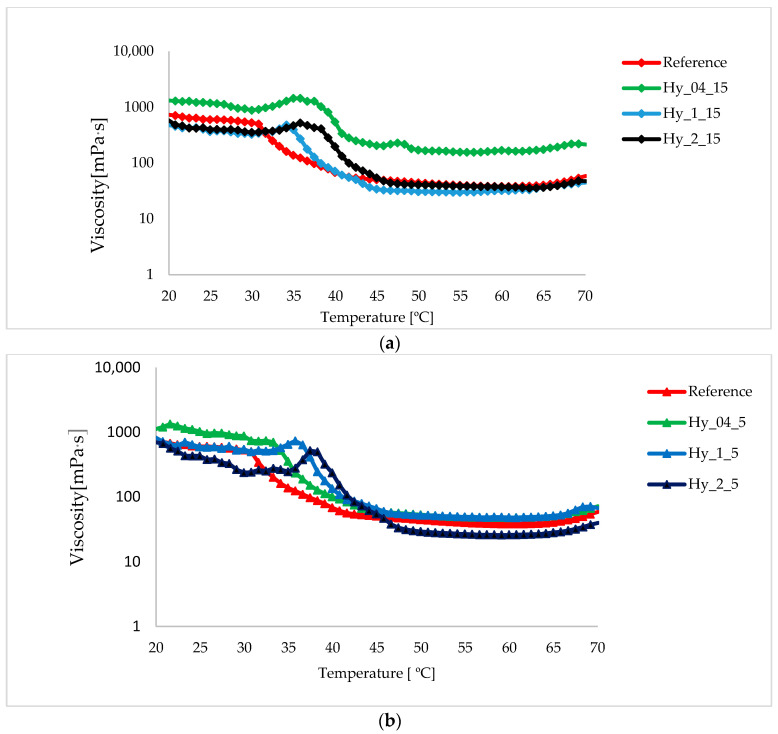
Influence of infusion duration (**a**)—5 min and (**b**)—10 min and the plant:water ratio of infusions on the viscosity of the 1% k carrageenan hydrogels during the temperature sweep test from 70 °C to 20 °C.

The determination of the solidification temperature (T_gel_) of the hydrogel was performed using a temperature ramp test, in which the solution was cooled from 70 °C to 20 °C. T_gel_ was determined as the point at which the viscosity was the highest, and was followed by a small drop, despite the decrease in temperature. T_gel_ was higher for the hydrogels obtained from 5 min infusions (Figure 3b). A more accurate method for the determination of Sol–gel transitions of the k-carrageenan aqueous solution is based on studying the dependence of tan δ as a function of temperature at different angular frequencies (Hz), which reported that the gelation temperature is 31.2 °C for 1% k carrageenan aqueous solution [32]. For the reference, the T_gel_ was determined at 30.76 °C, which is a similar value, whereas for the infusion containing hydrogels, T_gel_ occurred more quickly at 36.6 °C. The water-soluble compounds extracted by infusion potentiate the action of k-carrageenan, creating a gel at higher temperatures. However, only some samples—Hy_04_15, Hy_04_5, and Hy_1_5—exhibited higher viscosity than the reference obtained with water on the studied temperature profile (70 °C to 20 °C). At the beginning of the experiment, the viscosity of Hy_04_15 was the highest (215.05 ± 39.53 mPa*s) and was increased to 1315.35 ± 279.52 mPa*s. The reference exhibited a viscosity of 734.82 ± 53.46 at 20 °C.

### 2.4. FTIR

**Figure 4:**
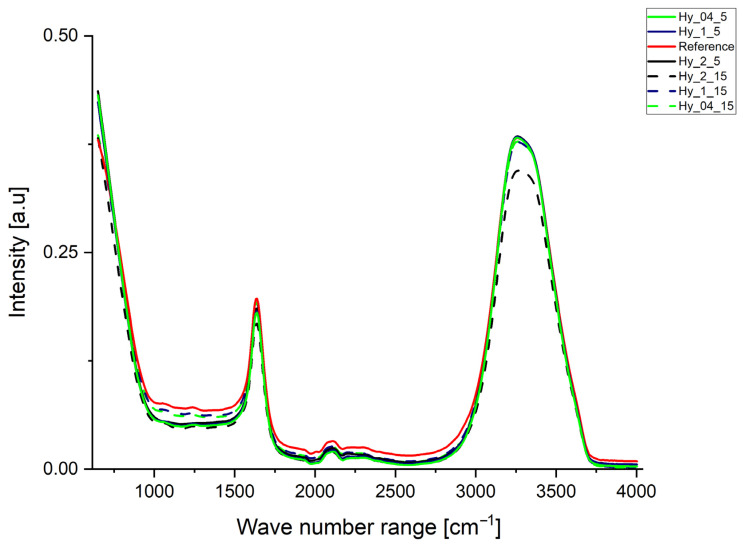
FTIR spectra (smoothed with Savitzky–Golay with 11 window and 2nd polynomial) of hydrogels obtained from infusion of *Urtica dioica* L.

The FTIR spectra of the hydrated hydrogels displayed peaks in the 3260–3300 cm^−1^ and the 1630–1640 cm^−1^ regions, due to a high water content (Figure 4). The FTIR spectra of the hydrogels obtained with k-carrageenan exhibited a small peak formed at 1235 cm^−1^, which is characteristic for S=O bonds, which are part of the sulfate ester groups of the k-carrageenan [33]. This might be due to the low amount of structuring agent involved in the formation of hydrogels (1%) or due to the hydrated state of the sample. The C=O stretching vibration from the organic acids extracted in the infusions led to the formation of peaks around 1630–1640 cm^−1^, while the peak exhibited by the reference is higher in intensity. Peaks at 3260–3300 cm^−1^ appeared because of the O-H bonds of the major compound of the hydrogels and the water, and could also be due to the C-H stretching vibrations of phenols [34]. However, we were unable to determine the relevant total polyphenol content for the hydrogel samples, except for Hy_2_15. Samples with an infusing duration of 5 min presented slightly higher intensities of the peaks in the 3260–3300 cm^−1^ region, while Hy_2_15 a peak with a lower intensity, highlighting the influence of the bioactive compounds that are extracted more efficiently, with the parameters being specific to the sample Hy_2_15 on the hydrogen bonding occurring in the polymer network formation.

### 2.5. Antioxidant Potential

The hydrogel obtained from the infusion with 2% nettle leaves and the infusion duration of 15 min exhibited the highest antioxidant potential values of 94.66% ± 0.49, followed by Hy_0.4_15, with the same infusing duration (highlighting the importance of ensuring the extraction of bioactive compounds in a longer time (15 min vs. 5 min). Hy_2_15 exhibited the highest TEAC of 662.33 ± 0.79 µmol TE/mL, followed by Hy_04_15.

**Figure 5:**
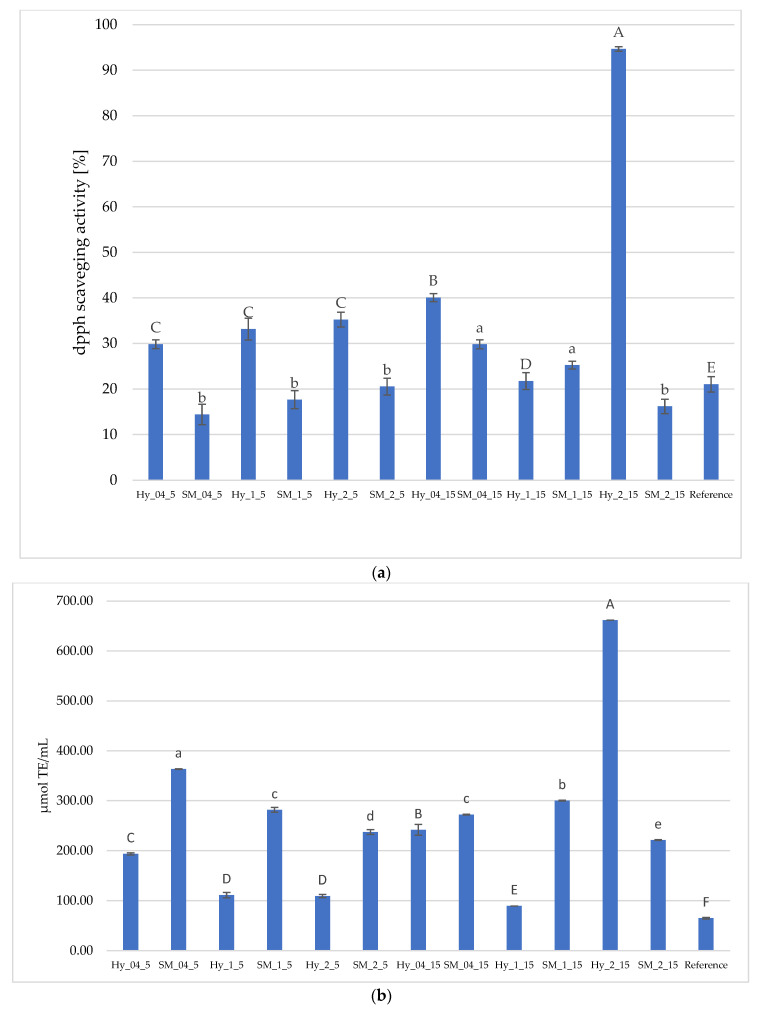
(**a**) DPPH scavenging activity of hydrogels (Hy) obtained from infusion of *Urtica dioica* L. with various plant:water ratio and that of the spent material (SM) separated after infusion preparation (**b**) antioxidant activities (TEAC) of the hydrogels (Hy) obtained from infusion of *Urtica dioica* L. with various plant:water ratio and the spent material (SM). Differences in capital letters indicate the statistical differences (*p* < 0.05) between the hydrogels, while small letters show statistical differences (*p* < 0.05) occurring between the spent materials. The results were presented as mean ± SD.

The rest of the samples exhibited moderate or low antioxidant potential, ranging between 21.74 and 40.08% DPPH scavenging activity [%] (Figure 5a). Hydrogels obtained with infusions with a 5 min duration exhibited statistically similar antioxidant potential, which was higher than the reference.

Hydrogels structured with carrageenan alone might exhibit antioxidant potential due to the hydroxyl groups in the polymer chain, while k-Carrageenan, which possesses lower molecular weights, exhibited relatively stronger antioxidant activities. The 1% k-carrageenan hydrogel showed a DPPH scavenging activity of 21.04 ± 1.70%.

Peppermint infusions prepared from dried leaves (1.5 g/100 g infused for 10 min) were reported to exhibit a DPPH scavenging activity of around 40%, while those from fresh leaves had percentages that were two times higher (88.61%) [35]. Hydrogel manufacturing could be explored by infusing the fresh leaves in subsequent studies.

Spent material properties are important, as it was reported that green tea can be brewed up to four times. SM_04_15 exhibited the highest DPPH scavenging activity [%] and TEAC among the spent materials, but was statistically similar to SM_1_15, whereas SM_2_15 had lower values, which were statistically similar to the rest of the samples (Figure 5b).

The spent material that showed a high antioxidant potential was also revealed by the ABTS assay, suggesting that a longer infusion duration, or the use of decoction as an extraction technique, could enhance the transfer of bioactive compounds in the aqueous extract.

## 3. Conclusions

Our findings demonstrate that replacing water with dried nettle leaf infusions significantly improves the gel stability, reducing syneresis rates, which is a major insufficiency of k-carrageenan hydrogels, especially in the first 24 h. Hydrogel manufacturing could be explored by infusing fresh leaves in subsequent studies. The hydrogels obtained from the dried nettle leaf infusions showed increased hardness, suggesting a modified polymer network, with higher storage moduli (G′) and elevated gelling temperatures than the water-based reference. The current study explored several plant-to-water ratios and infusion durations. The modification of the polymer network resulting from this variation was apparent in the FTIR spectra of the Hy_2_15. Among the formulations tested, Hy_2_15 emerged as the most promising, with the highest antioxidant activity (94.66% DPPH inhibited), followed by the remaining samples, including the reference. The synergy between the nettle compounds extracted from the infusion and the k-carrageenan may be responsible for the gel’s physical durability, while providing a high concentration of antioxidants. These results suggest that the plant-to-water ratio and infusing duration are critical parameters for tuning the rheological and biological potential of hydrogels. The infusing duration or plant-to-water ratio could be improved to achieve lower antioxidant potential in the spent material. Tuning the physical properties and biological efficacy of hydrogels by varying the infusing parameters ensures the feasibility for medical or food applications, including the formulation of emulgels or bigels, given the lower (<72) mechanical stability of the gels. Subsequent investigation would focus on the long-term storage stability of these bioactive-loaded systems to ensure the preservation of their antioxidant efficacy.

## 4. Materials and Methods

### 4.1. Plant Sample Collection

Plant samples were collected in May from the spontaneous flora near Gherla city, Cluj County, Romania, Latitude 47.03333000, Longitude 23.91667000. K-carragenan semi-refined was kindly offered by Solina, Alba Iulia, Romania. Methanol was purchased from Merck, Darmstadt, Germany. DPPH reagent was purchased from Sigma Aldrich, Darmstadt, Germany. ABTS (2,2′-Azino-bis(3-ethylbenzothiazoline-6-sulfonic acid) diammonium salt) and TROLOX were purchased from Alfa Aesar (Thermo Fisher Scientific, Karlsruhe, Germany) and ACROS organics (Thermo Fisher Scientific, Shanghai, China), respectively. All of the reagents were of an analytical grade.

### 4.2. Preparation of the Infusions

The leaves were washed and separated from their stems prior to drying (60 °C/24 h). The dried samples were placed in boiling water to obtain infusions at different plant: water ratios (0.4, 1 or 2 g/100 mL), with an infusion duration of 5 or 15 min.

### 4.3. Hydrogels Preparation

The obtained infusions were stabilized at 70 °C to prepare the hydrogels and 1% k-carrageenan was added; the mixtures were further stirred on a heated magnetic plate (70 °C) for 10 min, and then cooled to 20 °C.

### 4.4. The pH and Color Parameters of the Infusions

The pH of the infusions was measured with a HHI9814 Groline pH-meter (Hanna Instruments, Woonsocket, RI, USA). L*, a*, and b* color parameters were assessed with a NR200 (3NH, Shenzhen, China) portable colorimeter, and calibration was undertaken in a white environment.

### 4.5. Determination of the Antioxidant Potential

The antioxidant potential of the hydrogels and spent material was in the DPPH assay; 0.1 g of the hydrogel sample or residue was left overnight with 10 mL of methanol, then ultrasonicated, vacuum-filtered, syringe-filtered (0.45 µm) and analyzed. Then, 0.1 mL of the extract was mixed with DPPH solution (3.9 mL), kept in the dark at an ambient temperature, and the absorbance of the mixtures was recorded at 515 nm after exactly 30 min, against methanol as a blank [36].

ABTS assay was also performed. Trolox was used in concentrations from 25 to 600 µmol/L for the calibration curve preparation. The results were expressed in µmol, the Trolox equivalent (TE)/mL sample [37].

### 4.6. Texture Analysis of Hydrogels

The texture of the hydrogels was assessed using a TPA test, using CT3 Brookfield Texture Analyzer (Brookfield Engineering Labs, Middleboro, MA, USA), TA11/1000 geometry, with a 40% deformation of the samples (trigger force 0.05 N, test speed 1.00 mm/s). The textural attributes of the samples were given by the software of the device.

### 4.7. Syneresis Rate

A gravimetric method was applied to examine the syneresis rate of the hydrogels. The samples were kept at 25 °C and weighed after 24 h and 72 h. The syneresis rate was calculated as previously described [33].

### 4.8. Rheological Analysis—Amplitude Sweep and Temperature Ramp

A MCR 302 Anton-Paar Rheometer (GmbH, Graz, Austria) was used to evaluate the linear viscoelastic region (LVR) of the hydrogels and the non-destructive deformation in a small-amplitude dynamic testing (PP25 geometry, at a frequency of 1 Hz, temperature of 20 °C), with oscillatory stress varying from 0.01 to 100%. The power-law model was calculated after the decay of the storage modulus, with the following formula: G′ = Kγ − *n*, where γ is the strain amplitude, K = consistency constant and *n* = power-law exponent.

To analyze the gelling properties of the hydrogels, the base plates were initially heated at 70 °C, and the samples were placed on them and then cooled at a rate of 1 °C/min to 20 °C (ramp linear). Following the temperature ramp test, the gelation behavior was studied.

### 4.9. FTIR Analysis

Spectra of hydrated hydrogels were captured in the range of 400~4000 cm^−1^ by using a Fourier transform infrared spectrometer (Agilent Cary 630, Agilent Technologies, Chelmsford, MA, USA) equipped with an ATR Diamond sampling module, with a resolution of 4 cm^−1^ and 64 scan. The spectra were analyzed in OriginPro 2024 software (© 1991–2024 OriginLab Corporation, Northampton, MA, USA).

### 4.10. Statistical Analysis

Analyses were conducted at least in duplicate, while infusion manufacturing was performed in two replicates, with each sample replicate being homogenized prior to developing hydrogels. One-way and/or two-way (for infusions) analyses of variance (ANOVA) were performed in the OriginPro 2024 Software. The results are presented as mean ± SD. Differences between pairs of means were assessed on the basis of confidence intervals, using Tukey’s Honestly Significant Difference (HSD) test (*p* < 0.05). The normality was assessed with Leven’s test.

## Figures and Tables

**Table 1:** Infusing parameters: appearance/pH characterization.

Plant-to-Water Ratio g/100 g(A)	Infusing Duration (min)(B)	Sample Codification	pH/ 21 °C	L	a*	b*
0.4	5	04_5	7.47 ± 0.007	23.94 ± 0.30 Ba	10.19 ± 0.26 Bb	5.84 ± 0.48 Bb
1	5	1_5	7.68 ± 0.007	27.21 ± 0.78 Aa	5.09 ± 0.42 Cb	6.00 ± 0.26 Bb
2	5	2_5	7.68 ± 0.007	7.90 ± 1.10 Cb	13.41 ± 1.54 Aa	8.85 ± 0.69 Ab
0.4	15	04_15	7.64 ± 0.007	9.57 ± 0.91 Cb	13.01 ± 0.63 Aa	10.51 ± 0.38 Aa
1	15	1_15	7.86 ± 0.007	8.32 ± 1.07 Cb	10.01 ± 0.88 Ba	9.11 ± 0.41 Ba
2	15	2_15	7.89 ± 0.007	9.45 ± 1.68 Cb	10.78 ± 0.28 Bb	8.80 ± 0.71 Bb

Results are presented as mean ± SD. Different small letters indicate significant differences among levels of factor (A) ratio within the same level of factor brewing duration (B) (Tukey’s HSD, *p* < 0.05), while different capital letters indicate significant differences among the levels of factor (A) ratio within the same level of factor brewing duration (B) (Tukey’s HSD, *p* < 0.05).

**Table 2:** Syneresis rate and textural attributes of hydrogels.

Sample	Syneresis—24 h (%)	Syneresis—72 h (%)	Hardness [N]	Resilience	Cohesiveness	Gumminess [N]
Hy_04_5	4.60 ± 0.10 BC	10.56 ± 0.49 B	16.78 ± 1.08 BC	0.24 ± 0.01 C	0.38 ± 0.01 BC	6.58 ± 0.29 C
Hy_1_5	4.89 ± 0.49 BC	10.44 ± 2.89 B	21.52 ± 1.76 A	0.27 ± 0.05 C	0.39 ± 0.01 BC	8.72 ± 0.23 B
Hy_2_5	3.34 ± 0.03 C	10.79 ± 0.50 AB	19.33 ± 0.26 AB	0.18 ± 0.05 D	0.38 ± 0.05 BC	6.95 ± 0.33 C
Hy_04_15	6.67 ± 0.33 B	14.35 ± 2.46 A	16.74 ± 1.33 BC	0.33 ± 0.01 B	0.47 ± 0.03 B	10.29 ± 0.16 A
Hy_1_15	3.77 ± 0.03 C	9.66 ± 1.20 B	15.95 ± 0.54 C	0.12 ± 0.03 E	0.31 ± 0.02 C	4.33 ± 0.28 E
Hy_2_15	3.43 ± 0.78 C	9.15 ± 1.61 B	18.11 ± 1.10 BC	0.11 ± 0.01 E	0.36 ± 0.01 BC	5.10 ± 1.13 D
Reference	9.73 ± 1.56 A	12.53 ± 2.74 AB	15.60 ± 1.80 C	0.41 ± 0.04 A	0.64 ± 0.04 A	9.92 ± 0.66 AB

Results are presented as mean ± SD. Different capital letters indicate significant differences among samples as reported in (Tukey’s HSD, *p* < 0.05).

**Table 3:** The power-law model parameters for the hydrogels.

Sample	K	*n*	R
Hy_04_5	9.57 ± 1.38 BC	0.4139 ± 0.008 ABC	0.9715 ± 0.01 A
Hy_1_5	19.69 ± 1.59 A	0.3682 ± 0.01 CD	0.9736 ± 0.007 A
Hy_2_5	19.95 ± 2.51 A	0.3273 ± 0.004 D	0.943 ± 0.01 A
Hy_04_15	9.34 ± 1.05 BC	0.4168 ± 0.004 AB	0.9737 ± 0.01 A
Hy_1_15	15.44 ± 2.56 AB	0.3845 ± 0.02 D	0.9324 ± 0.007 A
Hy_2_15	16.19 ± 2.60 AB	0.3695 ± 0.006 BCD	0.9596 ± 0.006 A
Reference	7.28 ± 1.07 C	0.4564 ± 0.003 A	0.9672 ± 0.01 A

Data are expressed as mean ± SD. Differences in capital letters indicate the statistical differences (*p* < 0.05) between the parameters.

## Data Availability

The original contributions presented in this study are included in the article. Further inquiries can be directed to the corresponding author.
